# Supplementary material for: Effects of temperature dependent viscosity and thermal conductivity on natural convection flow along a curved surface in the presence of exothermic catalytic chemical reaction
Source: PLoS One. 2021 Jul 29;16(7):e0252485. doi: 10.1371/journal.pone.0252485 (PMC8321136; doi:10.1371/journal.pone.0252485)
Supplement: S1 Nomenclature — (DOCX) [file pone.0252485.s001.docx]

**S1 Nomenclature**

$n$ index parameter

Pr Prandtl number

Sc Schmidt number

$u$dimensionless velocity along $x$direction

$v$dimensionless velocity along y direction

x dimensionless distance along the surface

y dimensionless distance normal to the surface

T Dimensioned temperature

$T_{\infty}$ Ambient temperature

$E$ Dimensionless activation energy

**Greek Symbols**

$\theta$ Dimensionless temperature

$\emptyset$ Dimensionless mass concentration

$\beta$ Exothermic parameter

$\xi$ Thermal conductivity variation parameter

$\gamma$ Temperature relative parameter

$\gamma_{\mu}$ Viscosity variation parameter

$\lambda$ Chemical reaction rate constant
