# Supplementary material for: Effects of temperature dependent viscosity and thermal conductivity on natural convection flow along a curved surface in the presence of exothermic catalytic chemical reaction
Source: PLoS One. 2021 Jul 29;16(7):e0252485. doi: 10.1371/journal.pone.0252485 (PMC8321136; doi:10.1371/journal.pone.0252485)
Supplement: Attachment — Submitted filename: Response to Reviewers Comments.docx [file pone.0252485.s002.docx]

**Response to Reviewers Comments**

**(PONE-D-21-04681)**

Effects of Temperature Dependent Viscosity and Thermal Conductivity on Natural Convection Flow along a Curved Surface in the Presence of Exothermic Catalytic Chemical Reaction

**Reviewer #1:** The report presents the natural convection boundary layer flow of a viscous incompressible fluid with temperature-dependent viscosity and temperature-dependent thermal conductivity in the presence of exothermic catalytic chemical reaction along a curved surface. I recommend major revision due to the following facts.

1. In Eq. (3), the authors added (u^2/2x)n to the convective acceleration term.
   Comment: What does the new term means? How did you derive it.

**Response:** During dimensionalization of the momentum equation we have equation of the form

$u\frac{\partial u}{\partial x}+v\frac{\partial u}{\partial y}+\frac{u^{2}}{2}\frac{1}{g_{x}}\frac{dg_{x}}{dx}=\gamma_{\mu}\left( \frac{\partial\theta}{\partial y} \right)\left( \frac{\partial u}{\partial y} \right) +\left( 1+\gamma_{\mu}\theta\right)\left( \frac{\partial^{2}u}{\partial y^{2}} \right)+\theta+\phi$,

Here, as	$\frac{\boldsymbol{1}}{\bar{\boldsymbol{x}}}\left( P(\bar{x})+Q(\bar{x}) \right)=\frac{1}{g_{x}}\frac{\boldsymbol{d}\boldsymbol{g}_{\boldsymbol{x}}}{\boldsymbol{d}\bar{\boldsymbol{x}}}$

and	$P\left( \bar{x} \right)+Q\left( \bar{x} \right)=n$	and thus	$\frac{\boldsymbol{1}}{\bar{\boldsymbol{x}}}\left( n \right)=\frac{1}{g_{x}}\frac{\boldsymbol{d}\boldsymbol{g}_{\boldsymbol{x}}}{\boldsymbol{d}\bar{\boldsymbol{x}}}$

$u\frac{\partial u}{\partial x}+v\frac{\partial u}{\partial y}+\frac{u^{2}}{2x}n=\gamma_{\mu}\left( \frac{\partial\theta}{\partial y} \right)\left( \frac{\partial u}{\partial y} \right) +\left( 1+\gamma_{\mu}\theta\right)\left( \frac{\partial^{2}u}{\partial y^{2}} \right)+\theta+\phi$,

2. After Eq. (6), it was written that n = P(x) + Q(x). Whereas () is the wall temperature function and the () is the body shape function.

Comment: The authors refer Readers to check "Pop, I. and Takhar, H. S. Free convection from a curved surface. Journal of Applied Mathematics and Mechanics, 73 (1993), pp. 534 -539" for details.
Comment: This is not acceptable. Update the research methodology with the derivation of such momentum equation.

**Response:** The detail derivation of $n=P\left( x \right)+Q(x)$ is given in below steps:

Since we know that

$$\frac{\boldsymbol{d}}{\boldsymbol{dx}}\left( \boldsymbol{lnx} \right)\boldsymbol{=}\frac{\boldsymbol{1}}{\boldsymbol{x}}$$

and

$$\boldsymbol{d}\left( \boldsymbol{lnx} \right)\boldsymbol{=}\frac{\boldsymbol{1}}{\boldsymbol{x}}\boldsymbol{dx}$$

which implies that $\frac{\boldsymbol{1}}{\boldsymbol{d(lnx)}}\boldsymbol{=}\frac{\boldsymbol{x}}{\boldsymbol{dx}}$**. (1)**

Also consider $\frac{\boldsymbol{d}}{\boldsymbol{dx}}\left( \boldsymbol{ln}\boldsymbol{g}_{\boldsymbol{x}} \right)\boldsymbol{=}\frac{\boldsymbol{1}}{\boldsymbol{g}_{\boldsymbol{x}}}\frac{\boldsymbol{d}\boldsymbol{g}_{\boldsymbol{x}}}{\boldsymbol{dx}}$

which implies $\boldsymbol{d}\left( \boldsymbol{ln}\boldsymbol{g}_{\boldsymbol{x}} \right)\boldsymbol{=}\frac{\boldsymbol{1}}{\boldsymbol{g}_{\boldsymbol{x}}}\boldsymbol{d}\boldsymbol{g}_{\boldsymbol{x}}$**. (2)**

Now combining the above two equations (1) and (2), we have

$\frac{\boldsymbol{d ln}\boldsymbol{g}_{\boldsymbol{x}}}{\boldsymbol{d lnx}}\boldsymbol{=}\frac{\boldsymbol{x}}{\boldsymbol{g}_{\boldsymbol{x}}}\frac{\boldsymbol{d}\boldsymbol{g}_{\boldsymbol{x}}}{\boldsymbol{dx}}$.

Now using the non-dimensionless variables, we may write the above equations as

$$\frac{\boldsymbol{d ln}\boldsymbol{g}_{\boldsymbol{x}}}{\boldsymbol{d ln}\bar{\boldsymbol{x}}\boldsymbol{l}}\boldsymbol{=}\frac{\bar{\boldsymbol{x}}\boldsymbol{l}}{\boldsymbol{g}_{\boldsymbol{x}}}\frac{\boldsymbol{d}\boldsymbol{g}_{\boldsymbol{x}}}{\boldsymbol{d}\bar{\boldsymbol{x}}\boldsymbol{l}}$$

which implies that

$$\frac{\boldsymbol{d ln}\boldsymbol{g}_{\boldsymbol{x}}}{\boldsymbol{d ln}\bar{\boldsymbol{x}}\boldsymbol{l}}\boldsymbol{=}\frac{\bar{\boldsymbol{x}}}{\boldsymbol{g}_{\boldsymbol{x}}}\frac{\boldsymbol{d}\boldsymbol{g}_{\boldsymbol{x}}}{\boldsymbol{d}\bar{\boldsymbol{x}}}$$

and thus we have	$\frac{\boldsymbol{1}}{\bar{\boldsymbol{x}}}\frac{\boldsymbol{d ln}\boldsymbol{g}_{\boldsymbol{x}}}{\boldsymbol{d ln}\bar{\boldsymbol{x}}\boldsymbol{l}}\boldsymbol{=}\frac{\boldsymbol{1}}{\boldsymbol{g}_{\boldsymbol{x}}}\frac{\boldsymbol{d}\boldsymbol{g}_{\boldsymbol{x}}}{\boldsymbol{d}\bar{\boldsymbol{x}}}$	**(3)**

Also, we consider $\frac{\boldsymbol{d}}{\boldsymbol{dx}}\left( \boldsymbol{ln}\boldsymbol{T}_{\boldsymbol{w}} \right)\boldsymbol{=}\frac{\boldsymbol{1}}{\boldsymbol{T}_{\boldsymbol{w}}}\frac{\boldsymbol{d}\boldsymbol{T}_{\boldsymbol{w}}}{\boldsymbol{dx}}\boldsymbol{=0}$

which implies that $\boldsymbol{d ln}\boldsymbol{T}_{\boldsymbol{w}}\boldsymbol{=0}$

and thus, we can write	$\frac{\boldsymbol{d ln}\boldsymbol{T}_{\boldsymbol{w}}}{\boldsymbol{d ln}\bar{\boldsymbol{x}}\boldsymbol{l}}\boldsymbol{=0}$	**(4)**

Now from equation (3) and (4), we have

$\frac{\boldsymbol{1}}{\bar{\boldsymbol{x}}}\left( \frac{\boldsymbol{d ln}\boldsymbol{T}_{\boldsymbol{w}}}{\boldsymbol{d ln}\bar{\boldsymbol{x}}\boldsymbol{l}}\boldsymbol{+}\frac{\boldsymbol{d ln}\boldsymbol{g}_{\boldsymbol{x}}}{\boldsymbol{d ln}\bar{\boldsymbol{x}}\boldsymbol{l}} \right)\boldsymbol{=}\frac{\boldsymbol{1}}{\boldsymbol{g}_{\boldsymbol{x}}}\frac{\boldsymbol{d}\boldsymbol{g}_{\boldsymbol{x}}}{\boldsymbol{d}\bar{\boldsymbol{x}}}$

which can also be written as	$\frac{\boldsymbol{1}}{\bar{\boldsymbol{x}}}\left( \boldsymbol{P(}\bar{\boldsymbol{x}}\boldsymbol{)+Q(}\bar{\boldsymbol{x}}\boldsymbol{)} \right)\boldsymbol{=}\frac{\boldsymbol{1}}{\boldsymbol{g}_{\boldsymbol{x}}}\frac{\boldsymbol{d}\boldsymbol{g}_{\boldsymbol{x}}}{\boldsymbol{d}\bar{\boldsymbol{x}}}$	(5)

Here, $P(\bar{x})$, the wall temperature function and $Q(\bar{x})$ the body shape function, are defined as

$\boldsymbol{P}\left( \bar{\boldsymbol{x}} \right)\boldsymbol{=}\frac{\boldsymbol{d ln}\boldsymbol{T}_{\boldsymbol{w}}}{\boldsymbol{d ln}\bar{\boldsymbol{x}}\boldsymbol{l}}\boldsymbol{, Q}\left( \bar{\boldsymbol{x}} \right)\boldsymbol{=}\frac{\boldsymbol{d ln}\boldsymbol{g}_{\boldsymbol{x}}}{\boldsymbol{d ln}\bar{\boldsymbol{x}}\boldsymbol{l}}$ **.** **(6)**

3. Research question is needed to harmonize the contribution of the report to the body of knowledge.
Comment: Research questions are needed at the end of the introduction section. Research questions are needed. Note that the results in this report are typical answers to unknown questions. This is true because the manuscript provides some powerful answers to unknown questions. Note that the research questions must connect the title to the analysis of results, and conclusion. This would guide authors not to generate many results that are not consistent to provide insight. The author should update the manuscript with appropriate and relevant research questions at the end of the introduction section. This would guide the author to structure logical analysis of results. Logical questions are expected. This would help readers to link what is known in the literature with the novelty of this study.

**Response:** The overall objectives of this research are to develop the mathematical model to study and compare the different modes of coupling the exothermic and catalytic chemical reaction via momentum, energy and mass concentration equation. Model predictions are used to assess the effects of different parameters on conversion of exothermic catalytic chemical reaction at heated curve generated by tangential component of acceleration due to gravity. The proposed model is used as the guidelines for the selection of a suitable coupling to achieve the desired applications. A numerical technique Finite Difference Method in conjunction with primitive variable formulation is used to investigate the coupling of exothermic reaction with catalyst particles. Furthermore, parametric effects of heated wall and mass concentration along the curved surface are studied and highlighted graphically and as well as in tabular form.

4. The similarity variables presented as Eq. (7) are dimensional. The unit of the first, u, is m^0.5 while that of v is m^{-1/4}, and that of y is also dimensional. Hence, the variables used to non-dimenzionalize the governing equation are not appropriate.

**Response**: In the section of numerical analysis, we have used primitive variable formulation given as below:

$u=x^{\frac{1}{2}}U\left( X,Y \right), v=x^{\frac{-1}{4}}V\left( X,Y \right), x=X, y=x^{\frac{1}{4}}Y,$

$\theta=\Theta\left( X.Y \right), C=\Phi\left( X,Y \right).$

It is pertinent to mention that primitive variable are dimensionless, we use this transformation to get primitive form of partial differential equations, please see red terms in each equation

After using the aforementioned transformations, we have the following form of system of equations:

$$\frac{U}{2}+X\frac{\partial U}{\partial X}-\frac{Y}{4}\frac{\partial U}{\partial Y}+\frac{\partial V}{\partial Y}=0,$$

$$\left[ \frac{1}{2}+\frac{n}{2} \right]U^{2}+XU\frac{\partial U}{\partial X}+\left( V-\frac{YU}{4} \right)\frac{\partial U}{\partial Y}=\gamma_{\mu}\frac{\partial\Theta}{\partial Y}\frac{\partial U}{\partial Y}+\left( 1+\gamma_{\mu}\Theta\right)\frac{\partial^{2}U}{\partial Y^{2}}+\Theta+\Phi,$$

$XU\frac{\partial\Theta}{\partial X}+\left( V-\frac{YU}{4} \right)\frac{\partial\Theta}{\partial Y}=\frac{1}{Pr}\left[ \xi\left( \frac{\partial\Theta}{\partial Y} \right)^{2}+\left( 1+\xi\Theta\right)\frac{\partial^{2}\Theta}{\partial Y^{2}} \right]+\beta\lambda^{2}X^{1/2}\left( 1+n\gamma\Theta\right)exp\left( \frac{-E}{1+\gamma\theta} \right)\Theta$,

$$XU\frac{\partial\Phi}{\partial X}+\left( V-\frac{YU}{4} \right)\frac{\partial\Phi}{\partial Y}=\frac{1}{Sc}\frac{\partial^{2}\Phi}{\partial Y^{2}}+\lambda^{2}X^{1/2}\left( 1+n\gamma\Theta\right)exp\left( \frac{-E}{1+\gamma\theta} \right)\Phi.$$

**Reviewer #2: Recommendation: Minor revision**

Authors should revise the manuscript according to the following comments.
• Nomenclature is must.

**Response:** The nomenclature has been included in the revised manuscript.

• Some grammatical and typo mistakes are found in the manuscript.

**Response:** In the revised manuscript we have correct the grammatical issues.

• The governing differential equations describing the flow are non-linear. Is the solution obtained unique?

**Response:** Please if you focus on the graphs and boundary conditions

$$\boldsymbol{U}_{\boldsymbol{i,j}}\boldsymbol{=}0, V_{i,j}=0, \Theta_{i,j} =1, \Phi_{i,j}=1 at Y_{j}=0$$

$U_{i,j}\to0, \theta_{i,j}\to0, \Phi_{i.j}\to0 as Y_{j}\to\infty$

In these graphs and all other graphs given in this study, the solutions are satisfying by the boundary conditions, it is evident of the correctness of the obtained results.

• Specify the applications of considered physical model or geometry.

**Response:** The proposed model is used as the guidelines for the selection of a suitable coupling to achieve the desired applications. The curved shaped geometry is used to design many problems of civil engineering as pressure barrier.

• Introduction section should be made more concise to show previous work in the field. At present a lot of related researches are stated in the introduction. However, not sufficient analysis is presented. The authors should ask themselves: what are the problems with the presented researches? Why is the recent work needed? Hope these can improve the present work by following past articles.
• Effect of Joule heating on MHD non‐Newtonian fluid flow past an exponentially stretching curved surface
• Magneto hydrodynamic Cattaneo-Christov flow past a cone and a wedge with variable heat source/sink
• Heat and mass transfer in MHD Casson nanofluid flow past a stretching sheet with thermophoresis and Brownian motion.
• Effect of asymmetrical heat rise/fall on the film flow of magneto hydrodynamic hybrid Ferro fluid
• Simultaneous solutions for first order and second order slips on micro polar fluid flow across a convective surface in the presence of Lorentz force and variable heat source/sink.
• Effect of thermal radiation on MHD Casson fluid flow over an exponentially stretching curved sheet
• Influence of non-uniform heat source/sink on the three-dimensional magneto hydrodynamic Carreau fluid flow past a stretching surface with modified Fourier’s law
• MHD Carreau Fluid Flow Past a Melting Surface with Cattaneo-Christov Heat Flux
• Physical aspects on unsteady MHD‐free convective stagnation point flow of micro polar fluid over a stretching surface
• Influence of viscous dissipation on MHD flow of micro polar fluid over a slandering stretching surface with modified heat flux model
• A non‐Fourier heat flux model for magneto hydrodynamics micro polar liquid flow across a coagulated sheet
Note: I want to see the revised version of the manuscript.

**Response:** Most relevant papers are added to enrich introduction (see in introduction ref [23] - [30]) in the revised manuscript.
