# Supplementary material for: Effects of temperature dependent viscosity and thermal conductivity on natural convection flow along a curved surface in the presence of exothermic catalytic chemical reaction
Source: PLoS One. 2021 Jul 29;16(7):e0252485. doi: 10.1371/journal.pone.0252485 (PMC8321136; doi:10.1371/journal.pone.0252485)
Supplement: Attachment — Submitted filename: Response to Reviewers Comments.docx [file pone.0252485.s003.docx]

**Reply to Review PONE-D-21-04681R1
Effects of Temperature Dependent Viscosity and Thermal Conductivity on Natural Convection Flow along a Curved Surface in the Presence of Exothermic Catalytic Chemical Reaction PLOS ONE**

Reviewer #1: The report presents the natural convection boundary layer flow of a viscous incompressible fluid with temperature-dependent viscosity and temperature-dependent thermal conductivity in the presence of exothermic catalytic chemical reaction along a curved surface. I recommend minor revision due to the following facts.

**Q1**. In Eq. (3), the authors added (u^2/2x)n to the convective acceleration term.

Comment: What does the new term means? How did you derive it.

In the report, the derivation presented by the Authors is in isolation. You are expected to derive it as a convective acceleration of momentum equation.

**Response**: The detail derivation of momentum equation is given as below:

The dimensioned form of momentum equation in article is given as under

$u\frac{\partial u}{\partial x}+v\frac{\partial u}{\partial y}=\frac{1}{\rho}\frac{\partial}{\partial y}\left( \mu\frac{\partial u}{\partial y} \right)+g_{x}\beta_{T}\left( T-T_{\infty} \right)+g_{x}\beta_{C}(C-C_{\infty})$	(1)

Dimensionless variables

$\bar{x}=\frac{x}{l}, \bar{y}=\frac{y}{l}Gr^{\frac{1}{4}},\bar{u}=\frac{u}{U_{s}}, \bar{v}=\frac{v}{V_{s}}Gr^{\frac{1}{4}},\theta=\frac{T-T_{\infty}}{T_{w}-T_{\infty}} ,\phi=\frac{C-C_{\infty}}{C_{w}-C_{\infty}}$	(2)

To convert the equation (1) into dimensionless form we use the dimensionless variables defined in equation (2), for this we find the term s one by one appeared in the equation (1)

$$u\frac{\partial u}{\partial x}=U_{s}\bar{u}\frac{\partial U_{s}\bar{u}}{\partial\bar{x}l}=\frac{U_{s}^{2}}{l}\bar{u}\frac{\partial\bar{u}}{\partial\bar{x}}+\frac{{U_{s}\bar{u}}^{2}}{l}\frac{\partial U_{s}}{\partial\bar{x}}$$

$$u\frac{\partial u}{\partial x}=\frac{U_{s}^{2}}{l}\bar{u}\frac{\partial\bar{u}}{\partial\bar{x}}+\frac{{U_{s}\bar{u}}^{2}}{l}\frac{\partial}{\partial\bar{x}}\left( g_{x}\beta_{T}\Delta T \right)^{\frac{1}{2}}$$

$$u\frac{\partial u}{\partial x}=\frac{U_{s}^{2}}{l}\bar{u}\frac{\partial\bar{u}}{\partial\bar{x}}+\frac{{U_{s}\bar{u}}^{2}}{l}\left( g_{x}\beta_{T}\Delta T \right)^{\frac{1}{2}}\frac{1}{2\sqrt{g_{x}}} \frac{dg_{x}}{d\bar{x}}$$

$u\frac{\partial u}{\partial x}=\frac{U_{s}^{2}}{l}\left[ \bar{u}\frac{\partial\bar{u}}{\partial\bar{x}}+\frac{\bar{u}^{2}}{2}\frac{1}{g_{x}} \frac{dg_{x}}{d\bar{x}} \right]$	(3)

Now

$v\frac{\partial u}{\partial y}=U_{s}\bar{v}Gr^{-\frac{1}{4}}\frac{\partial U_{s}\bar{u}}{\partial l\bar{y}Gr^{-\frac{1}{4}}}=\frac{U_{s}^{2}}{l}\bar{v}\frac{\partial\bar{u}}{\partial\bar{y}}$	(4)

Now

$\frac{1}{\rho} \frac{\partial}{\partial y}\left( \mu\frac{\partial u}{\partial y} \right)=\frac{1}{\rho}\left[ \frac{\partial\mu}{\partial y}\frac{\partial u}{\partial y}+\mu\frac{\partial^{2}u}{\partial y^{2}} \right]$	(5)

Considering

$$\frac{\partial\mu}{\partial y}=\frac{\partial}{\partial y}\mu_{o}(1+\gamma^{*}(T-T_{\infty}))$$

$$\frac{\partial\mu}{\partial y}=\frac{\partial}{\partial\bar{y}lGr^{-\frac{1}{4}}}\left( \mu_{o}(1+\gamma^{*}\Delta T\theta) \right)$$

$$\frac{\partial\mu}{\partial y}=\frac{\mu_{o}\gamma^{*}\Delta T}{lGr^{-\frac{1}{4}}}\frac{\partial\theta}{\partial\bar{y}}$$

$\frac{\partial\mu}{\partial y}=\frac{\mu_{o}\gamma_{T}}{lGr^{-\frac{1}{4}}}\frac{\partial\theta}{\partial\bar{y}}$	(6)

$\frac{\partial u}{\partial y}=\frac{\partial U_{s}\bar{u}}{\partial\bar{y}lGr^{-\frac{1}{4}}}=\frac{U_{s}}{lGr^{-\frac{1}{4}}}\frac{\partial\bar{u}}{\partial\bar{y}}$	(7)

$\frac{\partial^{2}u}{\partial y^{2}}=\frac{\partial U_{s}\bar{u}}{\partial\bar{y}lGr^{-\frac{1}{4}}}=\frac{U_{s}}{lGr^{-\frac{1}{2}}}\frac{\partial^{2}\bar{u}}{\partial\bar{y}^{2}}$	(8)

Put eqs. (6)-(8) in eq. (5) we have

$$\frac{1}{\rho} \frac{\partial}{\partial y}\left( \mu\frac{\partial u}{\partial y} \right)=\frac{1}{\rho}\left[ \frac{\mu_{o}\gamma_{\mu}}{lGr^{-\frac{1}{4}}}\frac{\partial\theta}{\partial\bar{y}}\frac{U_{s}}{lGr^{-\frac{1}{4}}}\frac{\partial\bar{u}}{\partial\bar{y}}+\frac{\mu U_{s}}{lGr^{-\frac{1}{2}}}\frac{\partial^{2}\bar{u}}{\partial\bar{y}^{2}} \right]$$

$$\frac{1}{\rho} \frac{\partial}{\partial y}\left( \mu\frac{\partial u}{\partial y} \right)=\frac{1}{\rho}\left[ \frac{\mu_{o}\gamma_{\mu}U_{s}}{l^{2}Gr^{-\frac{1}{2}}}\frac{\partial\theta}{\partial\bar{y}}\frac{\partial\bar{u}}{\partial\bar{y}}+\frac{{\mu_{o}(1+\gamma_{\mu}\theta)U}_{s}}{l^{2}Gr^{-\frac{1}{2}}}\frac{\partial^{2}\bar{u}}{\partial\bar{y}^{2}} \right]$$

$$\frac{1}{\rho} \frac{\partial}{\partial y}\left( \mu\frac{\partial u}{\partial y} \right)=\frac{\mu_{o}U_{s}}{\rho l^{2}Gr^{-\frac{1}{2}}}\left[ \gamma_{\mu}\frac{\partial\theta}{\partial\bar{y}}\frac{\partial\bar{u}}{\partial\bar{y}}+(1+\gamma_{\mu}\theta)\frac{\partial^{2}\bar{u}}{\partial\bar{y}^{2}} \right]$$

Where $\gamma_{\mu=}\gamma^{*}\Delta T$

$\frac{1}{\rho} \frac{\partial}{\partial y}\left( \mu\frac{\partial u}{\partial y} \right)=\frac{\mu_{o}U_{s}}{\rho l^{2}Gr^{-\frac{1}{2}}}\left[ \gamma_{\mu}\frac{\partial\theta}{\partial\bar{y}}\frac{\partial\bar{u}}{\partial\bar{y}}+(1+\gamma_{\mu}\theta)\frac{\partial^{2}\bar{u}}{\partial\bar{y}^{2}} \right]$	(9)

Put the eqs. (3-4) and Eq. (9) in eq. (1)

$\frac{U_{s}^{2}}{l}\left[ \bar{u}\frac{\partial\bar{u}}{\partial\bar{x}}+\frac{\bar{u}^{2}}{2}\frac{1}{g_{x}} \frac{dg_{x}}{d\bar{x}}+\bar{v}\frac{\partial\bar{u}}{\partial\bar{y}} \right]=\frac{\mu_{o}U_{s}Gr^{\frac{1}{2}}}{\rho l^{2}}\left[ \gamma_{\mu}\frac{\partial\theta}{\partial\bar{y}}\frac{\partial\bar{u}}{\partial\bar{y}}+(1+\gamma_{\mu}\theta)\frac{\partial^{2}\bar{u}}{\partial\bar{y}^{2}} \right]+g_{x}\beta_{T}\Delta T\theta+g_{x}\beta_{C}\Delta C\phi$

$\left[ \bar{u}\frac{\partial\bar{u}}{\partial\bar{x}}+\frac{\bar{u}^{2}}{2}\frac{1}{g_{x}} \frac{dg_{x}}{d\bar{x}}+\bar{v}\frac{\partial\bar{u}}{\partial\bar{y}} \right]=\frac{\mu_{o}Gr^{\frac{1}{2}}}{\rho lU_{s}}\left[ \gamma_{\mu}\frac{\partial\theta}{\partial\bar{y}}\frac{\partial\bar{u}}{\partial\bar{y}}+(1+\gamma_{\mu}\theta)\frac{\partial^{2}\bar{u}}{\partial\bar{y}^{2}} \right]+\frac{{lg}_{x}\beta_{T}\Delta T\theta}{U_{s}^{2}}+\frac{{lg}_{x}\beta_{C}\Delta T\phi}{U_{s}^{2}}$

$$\bar{u}\frac{\partial\bar{u}}{\partial\bar{x}}+\frac{\bar{u}^{2}}{2}\frac{1}{g_{x}} \frac{dg_{x}}{d\bar{x}}+\bar{v}\frac{\partial\bar{u}}{\partial\bar{y}}=\frac{\nu Gr^{\frac{1}{2}}}{lU_{s}}\left[ \gamma_{\mu}\frac{\partial\theta}{\partial\bar{y}}\frac{\partial\bar{u}}{\partial\bar{y}}+(1+\gamma_{\mu}\theta)\frac{\partial^{2}\bar{u}}{\partial\bar{y}^{2}} \right]+\frac{{lg}_{x}\beta_{T}\Delta T\theta}{U_{s}^{2}}+\frac{{lg}_{x}\beta_{C}\Delta C\phi}{U_{s}^{2}}$$

$$\bar{u}\frac{\partial\bar{u}}{\partial\bar{x}}+\frac{\bar{u}^{2}}{2}\frac{1}{g_{x}} \frac{dg_{x}}{d\bar{x}}+\bar{v}\frac{\partial\bar{u}}{\partial\bar{y}}=\frac{\nu Gr^{\frac{1}{2}}}{lU_{s}}\left[ \gamma_{\mu}\frac{\partial\theta}{\partial\bar{y}}\frac{\partial\bar{u}}{\partial\bar{y}}+(1+\gamma_{\mu}\theta)\frac{\partial^{2}\bar{u}}{\partial\bar{y}^{2}} \right]+\frac{{lg}_{x}\beta_{T}\Delta T\theta}{U_{s}^{2}}+\frac{{lg}_{x}\beta_{C}\Delta C\phi}{U_{s}^{2}}$$

Where $U_{s}=\left( g_{x}\beta_{T}\Delta Tl \right)^{\frac{1}{2}}, Gr=\frac{g_{x}\beta_{T}\Delta Tl^{3}}{\nu^{2}}$ and $U_{sc}=\left( g_{x}\beta_{T}\Delta Cl \right)^{\frac{1}{2}}, Gr^{*}=\frac{g_{x}\beta_{T}\Delta Cl^{3}}{\nu^{2}}$

So

$$\bar{u}\frac{\partial\bar{u}}{\partial\bar{x}}+\frac{\bar{u}^{2}}{2}\frac{1}{g_{x}} \frac{dg_{x}}{d\bar{x}}+\bar{v}\frac{\partial\bar{u}}{\partial\bar{y}}=\frac{\nu Gr^{\frac{1}{2}}}{lU_{s}}\left[ \gamma_{\mu}\frac{\partial\theta}{\partial\bar{y}}\frac{\partial\bar{u}}{\partial\bar{y}}+(1+\gamma_{\mu}\theta)\frac{\partial^{2}\bar{u}}{\partial\bar{y}^{2}} \right]+\frac{U_{s}^{2}\theta}{U_{s}^{2}}+\frac{U_{s}^{2}\phi}{U_{s}^{2}}$$

$\bar{u}\frac{\partial\bar{u}}{\partial\bar{x}}+\frac{\bar{u}^{2}}{2}\frac{1}{g_{x}} \frac{dg_{x}}{d\bar{x}}+\bar{v}\frac{\partial\bar{u}}{\partial\bar{y}}=\gamma_{\mu}\frac{\partial\theta}{\partial\bar{y}}\frac{\partial\bar{u}}{\partial\bar{y}}+(1+\gamma_{\mu}\theta)\frac{\partial^{2}\bar{u}}{\partial\bar{y}^{2}}+\theta+\phi$	(10)

Where the detail derivation of $n=P\left( x \right)+Q(x)$ is given in below steps:

Since we know that

$$\frac{\boldsymbol{d}}{\boldsymbol{dx}}\left( \boldsymbol{lnx} \right)\boldsymbol{=}\frac{\boldsymbol{1}}{\boldsymbol{x}}$$

and

$$\boldsymbol{d}\left( \boldsymbol{lnx} \right)\boldsymbol{=}\frac{\boldsymbol{1}}{\boldsymbol{x}}\boldsymbol{dx}$$

which implies that$\frac{\boldsymbol{1}}{\boldsymbol{d}\boldsymbol{(}\boldsymbol{lnx}\boldsymbol{)}}\boldsymbol{=}\frac{\boldsymbol{x}}{\boldsymbol{dx}}$**.**

Also consider$\frac{\boldsymbol{d}}{\boldsymbol{dx}}\left( \boldsymbol{ln} \boldsymbol{g}_{\boldsymbol{x}} \right)\boldsymbol{=}\frac{\boldsymbol{1}}{\boldsymbol{g}_{\boldsymbol{x}}}\frac{\boldsymbol{d}\boldsymbol{g}_{\boldsymbol{x}}}{\boldsymbol{dx}}$

which implies$\boldsymbol{d}\left( \boldsymbol{ln} \boldsymbol{g}_{\boldsymbol{x}} \right)\boldsymbol{=}\frac{\boldsymbol{1}}{\boldsymbol{g}_{\boldsymbol{x}}}\boldsymbol{d}\boldsymbol{g}_{\boldsymbol{x}}$**.**

$\frac{\boldsymbol{d} \boldsymbol{ln}\boldsymbol{g}_{\boldsymbol{x}}}{\boldsymbol{d} \boldsymbol{lnx}}\boldsymbol{=}\frac{\boldsymbol{x}}{\boldsymbol{g}_{\boldsymbol{x}}}\frac{\boldsymbol{d}\boldsymbol{g}_{\boldsymbol{x}}}{\boldsymbol{dx}}$.

Now using the non-dimensionless variables, we may write the above equations as

$$\frac{\boldsymbol{d} \boldsymbol{ln}\boldsymbol{g}_{\boldsymbol{x}}}{\boldsymbol{d} \boldsymbol{ln}\bar{\boldsymbol{x}}\boldsymbol{l}}\boldsymbol{=}\frac{\bar{\boldsymbol{x}}\boldsymbol{l}}{\boldsymbol{g}_{\boldsymbol{x}}}\frac{\boldsymbol{d}\boldsymbol{g}_{\boldsymbol{x}}}{\boldsymbol{d}\bar{\boldsymbol{x}}\boldsymbol{l}}$$

which implies that

$$\frac{\boldsymbol{d} \boldsymbol{ln}\boldsymbol{g}_{\boldsymbol{x}}}{\boldsymbol{d} \boldsymbol{ln}\bar{\boldsymbol{x}}\boldsymbol{l}}\boldsymbol{=}\frac{\bar{\boldsymbol{x}}}{\boldsymbol{g}_{\boldsymbol{x}}}\frac{\boldsymbol{d}\boldsymbol{g}_{\boldsymbol{x}}}{\boldsymbol{d}\bar{\boldsymbol{x}}}$$

and thus we have $\frac{\boldsymbol{1}}{\bar{\boldsymbol{x}}}\frac{\boldsymbol{d} \boldsymbol{ln}\boldsymbol{g}_{\boldsymbol{x}}}{\boldsymbol{d} \boldsymbol{ln}\bar{\boldsymbol{x}}\boldsymbol{l}}\boldsymbol{=}\frac{\boldsymbol{1}}{\boldsymbol{g}_{\boldsymbol{x}}}\frac{\boldsymbol{d}\boldsymbol{g}_{\boldsymbol{x}}}{\boldsymbol{d}\bar{\boldsymbol{x}}}$

Also, we consider $\frac{\boldsymbol{d}}{\boldsymbol{dx}}\left( \boldsymbol{ln}\boldsymbol{T}_{\boldsymbol{w}} \right)\boldsymbol{=}\frac{\boldsymbol{1}}{\boldsymbol{T}_{\boldsymbol{w}}}\frac{\boldsymbol{d}\boldsymbol{T}_{\boldsymbol{w}}}{\boldsymbol{dx}}\boldsymbol{=}\boldsymbol{0}$

which implies that $\boldsymbol{d} \boldsymbol{ln}\boldsymbol{T}_{\boldsymbol{w}}\boldsymbol{=}\boldsymbol{0}$

and thus, we can write $\frac{\boldsymbol{d} \boldsymbol{ln}\boldsymbol{T}_{\boldsymbol{w}}}{\boldsymbol{d} \boldsymbol{ln}\bar{\boldsymbol{x}}\boldsymbol{l}}\boldsymbol{=}\boldsymbol{0}$

Now from equation (c) and (d), we have

$$\frac{\boldsymbol{1}}{\bar{\boldsymbol{x}}}\left( \frac{\boldsymbol{d} \boldsymbol{ln}\boldsymbol{T}_{\boldsymbol{w}}}{\boldsymbol{d} \boldsymbol{ln}\bar{\boldsymbol{x}} \boldsymbol{l}}\boldsymbol{+}\frac{\boldsymbol{d} \boldsymbol{ln}\boldsymbol{g}_{\boldsymbol{x}}}{\boldsymbol{d} \boldsymbol{ln}\bar{\boldsymbol{x}} \boldsymbol{l}} \right)\boldsymbol{=}\frac{\boldsymbol{1}}{\boldsymbol{g}_{\boldsymbol{x}}}\frac{\boldsymbol{d}\boldsymbol{g}_{\boldsymbol{x}}}{\boldsymbol{d}\bar{\boldsymbol{x}}}$$

which can also be written as $\frac{\boldsymbol{1}}{\bar{\boldsymbol{x}}}\left( \boldsymbol{P}\boldsymbol{(}\bar{\boldsymbol{x}}\boldsymbol{)+}\boldsymbol{Q}\boldsymbol{(}\bar{\boldsymbol{x}}\boldsymbol{)} \right)\boldsymbol{=}\frac{\boldsymbol{1}}{\boldsymbol{g}_{\boldsymbol{x}}}\frac{\boldsymbol{d}\boldsymbol{g}_{\boldsymbol{x}}}{\boldsymbol{d}\bar{\boldsymbol{x}}}$

Here, $P(\bar{x})$, the wall temperature function and $Q(\bar{x})$ the body shape function, are defined as

$\boldsymbol{P}\left( \bar{\boldsymbol{x}} \right)\boldsymbol{=}\frac{\boldsymbol{d} \boldsymbol{ln}\boldsymbol{T}_{\boldsymbol{w}}}{\boldsymbol{d} \boldsymbol{ln}\bar{\boldsymbol{x}} \boldsymbol{l}}\boldsymbol{,}\boldsymbol{Q}\left( \bar{\boldsymbol{x}} \right)\boldsymbol{=}\frac{\boldsymbol{d} \boldsymbol{ln}\boldsymbol{g}_{\boldsymbol{x}}}{\boldsymbol{d} \boldsymbol{ln}\bar{\boldsymbol{x}} \boldsymbol{l}}$ **.**

Now (10) becomes

$\bar{u}\frac{\partial\bar{u}}{\partial\bar{x}}+\frac{\bar{u}^{2}}{2}(P\left( \bar{x} \right)+Q(\bar{x}))+\bar{v}\frac{\partial\bar{u}}{\partial\bar{y}}=\gamma_{\mu}\frac{\partial\theta}{\partial\bar{y}}\frac{\partial\bar{u}}{\partial\bar{y}}+(1+\gamma_{\mu}\theta)\frac{\partial^{2}\bar{u}}{\partial\bar{y}^{2}}+\theta+\phi$	(11)

Now $P\left( \bar{x} \right)+Q\left( \bar{x} \right)=n$

Thus (11) becomes

$\bar{u}\frac{\partial\bar{u}}{\partial\bar{x}}+\frac{\bar{u}^{2}}{2}\frac{1}{g_{x}} \frac{dg_{x}}{d\bar{x}}+\bar{v}\frac{\partial\bar{u}}{\partial\bar{y}}=\gamma_{\mu}\frac{\partial\theta}{\partial\bar{y}}\frac{\partial\bar{u}}{\partial\bar{y}}+(1+\gamma_{\mu}\theta)\frac{\partial^{2}\bar{u}}{\partial\bar{y}^{2}}+\theta+\phi$

Which is dimensionless form of momentum equation along curved surface, and $n$ is the body shape parameter.

**Q2**. After Eq. (6), it was written that n = P(x) + Q(x). Whereas () is the wall temperature function and
the () is the body shape function. This is the exact contribution of the report to the body of knowledge and it should be introduced under the introduction section. In fact, a paragraph is needed to announce this novelty.

**Response:** In current paper we are investigated the natural convection flow over a two dimensional body of arbitrary geometric configuration in the presence of exothermic catalytic chemical reaction. The momentum, energy, and mass concentration equations are a general form suitable for laminar natural convection flows along curved surface in the inclusion of exothermic catalytic reaction. For body of arbitrary shape, the special case in which $P\left( \bar{x} \right)+Q\left( \bar{x} \right)=n$, thus the body shape parameter (index parameter) $n$ has chosen $0<n\leq\frac{1}{2}$ which is main novelty of this work.

Note: This paragraph has inserted in the introduction section.

**Q3**. How do you study a fluid flow along a vertical surface without buoyancy forces? This is true because there is nothing like the associated dimensionless parameter called buoyancy parameter or Grashof number.

**Response:** Please if you see the dimensionless variables used to dimensionalize the flow model

Dimensionless variables

$\bar{x}=\frac{x}{l}, \bar{y}=\frac{y}{l}Gr^{\frac{1}{4}},\bar{u}=\frac{u}{U_{s}}, \bar{v}=\frac{v}{V_{s}}Gr^{\frac{1}{4}}, \theta=\frac{T-T_{\infty}}{T_{w}-T_{\infty}} ,\phi=\frac{C-C_{\infty}}{C_{w}-C_{\infty}}$

(i) Here Grashof number is used as $\bar{y}=\frac{y}{l}Gr^{\frac{1}{4}}$, $\bar{v}=\frac{v}{V_{s}}Gr^{\frac{1}{4}}$ where $Gr=\frac{g\beta\Delta Tx^{3}}{\nu^{2}}$, which is infact responsible for buoyancy force and it is used in dimensionalization (frequently available in literature (you can see Convective Heat Transfer by Ion Pop and D. B. Ingham for the cases of free or natural convection heat transfer).
(ii) Dimensionless temperature variable $\theta=\frac{T-T_{\infty}}{T_{w}-T_{\infty}}$, it is the ratio of temperature difference. The problem under consideration is based on natural convection heat transfer, natural convection occurred because of the difference in density, and difference in density is due to the difference in temperature. The contribution of $\theta$ in momentum is obvious and sufficient answer to question.

**Q4.** Before Eq. 7, was defined as the tangential component of acceleration due to gravity. What is r in the definition?

**Response:** Here, $x$ is the boundary layer coordinate along the curved surface while $r$ and $z$ are the horizontal and vertical coordinates of the points of the shape curve $z=z(r)$ respectively. It is pertinent to mention that here flow is along the curved surface and $\frac{dr}{dz}=0$ means there is no flow inner side of the curve. The coordinate $z$ is the vertical distance measure from the lower stagnation point (Please see fig. 1 flow configuration and coordinate system).

Reviewer #2: The revisions are Good but some references are not arranged properly. Hence I recommend for possible publication.

**Response**: We have focused on references, all the references are now in good order and well targeted to the problem under consideration.
